# Chemically Propelled Motors Navigate Chemical Patterns

**DOI:** 10.1002/advs.201800028

**Published:** 2018-07-11

**Authors:** Jiang‐Xing Chen, Yu‐Guo Chen, Raymond Kapral

**Affiliations:** ^1^ Department of Physics Hangzhou Dianzi University Hangzhou 310018 China; ^2^ Chemical Physics Theory Group Department of Chemistry University of Toronto Toronto Ontario M5S 3H6 Canada

**Keywords:** active self‐assembly, collective motor dynamics, controlled motions of motors along prescribed paths, far‐from‐equilibrium active chemical media

## Abstract

Very small synthetic motors that use chemical reactions to drive their motion are being studied widely because of their potential applications, which often involve active transport and dynamics on nanoscales. Like biological molecular machines, they must be able to perform their tasks in complex, highly fluctuating environments that can form chemical patterns with diverse structures. Motors in such systems can actively assemble into dynamic clusters and other unique nonequilibrium states. It is shown how chemical patterns with small characteristic dimensions may be utilized to suppress rotational Brownian motions of motors and guide them to move along prescribed paths, properties that can be exploited in applications. In systems with larger pattern length scales, domains can serve as catch basins for motors through chemotactic effects. The resulting collective motor dynamics in such confining domains can be used to explore new aspects of active particle collective dynamics or promote specific types of active self‐assembly. More generally, when chemically self‐propelled motors operate in far‐from‐equilibrium active chemical media the variety of possible phenomena and the scope of their potential applications are substantially increased.

## 1 Introduction

Similar to biological molecular machines in the cell,^1^, ^2^, ^3^ very small synthetic chemically powered motors are able to extract chemical energy from fuel in their environment and convert it into directed motion. Unlike molecular machines, such motors often have no moving parts and do not rely on conformational changes for their motion.^4^ Instead they make use of asymmetric catalytic activity on the motor surface to produce propulsion.^5^, ^6^, ^7^, ^8^ Like molecular machines, they operate out of equilibrium and their motion is strongly influenced by thermal fluctuations in the surrounding medium.

These micro‐ and nanoscale motors are able to actively transport material and perform other tasks on small scales. Since these small motors experience strong thermal fluctuations, rotational Brownian motion changes their propagation direction giving rise to enhanced diffusion on long time scales. In order to fully exploit the directed motion of these motors for applications one must be able to mitigate the effects of these orientational fluctuations. Biological molecular machines achieve this by confining their motion to “walking” on filaments.^9^ A similar strategy could be proposed by synthetic chemically powered motors.^10^ Alternatively, external fields^8^, ^11^, ^12^, ^13^, ^14^, ^15^ and interactions with surfaces^16^, ^17^, ^18^, ^19^ have also been proposed as ways to control motor motion. When many motors interact with each other, phenomena such as dynamic clustering, swarming and active phase segregation are observed.^20^, ^21^, ^22^, ^23^, ^24^ These features have stimulated research on the design and properties of motors that is aimed at potential applications.^5^, ^12^, ^25^, ^26^, ^27^, ^28^, ^29^, ^30^, ^31^

The fuel that motors use for propulsion may be introduced into the system by fluxes of chemical species at the boundaries or, as is the case for many biological systems, by complex networks of out‐of‐equilibrium chemical reactions within the surrounding medium. Spatially distributed reaction networks can support a variety of different spatiotemporal nonequilibrium states, including target patterns, spiral waves and other stationary inhomogeneous states such as labyrinthine patterns and linked and knotted chemical domains.^32^, ^33^ Turing patterns in the form of stripes and spots,^34^ and domains in bistable chemical media with competing interactions^35^ have been observed in early laboratory experiments. Alternatively, the chemical patterns could be produced by fabricating domains of catalyst on a surface that promote the production of fuel from reactions in the medium.^36^ Chemical patterns may have macroscopic to nanoscopic characteristic lengths, allowing systems with a wide range of length scales to be explored.^37^, ^38^, ^39^, ^40^, ^41^ When chemically propelled motors operate in such media new phenomena appear; for example, it has been shown that chemically propelled nanomotors can be reflected from traveling chemical fronts in media where cubic autocatalytic reactions take place.^42^

In this paper, we study the dynamics of synthetic motors propelled by a self‐diffusiophoretic mechanism using the hybrid molecular dynamics‐multiparticle collision dynamics (MD‐MPC) method. The dimer motors are powered by surface reactions involving chemical species in the surrounding medium that exists in a chemically patterned state. Both single motor motion and the collective motion of small ensembles of motors are considered. Such motors are able to respond to gradients in fuel or product molecules, and show various types of dynamic behavior, for example, the chemotactic behavior seen in systems of self‐propelled swimmers.^43^, ^44^, ^45^, ^46^, ^47^ Therefore, chemical patterns that involve gradients in these species concentrations can influence the motions of motors. Consequently, when chemical domains in far‐from‐equilibrium systems possess characteristic lengths comparable to those of the motor, reacting media may be used to guide motor motion. Larger domains can act as sinks that trap motors promoting specific types of active self‐assembly that may aid in the design of new materials.

## 2 Results and Discussion

**Figure 1:**
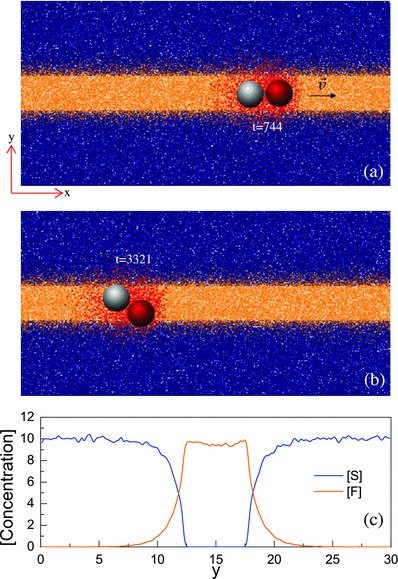
a) Instantaneous configuration at *t* = 744 shows a dimer moving in a striped chemical pattern. The arrow indicates the direction of motion. Species F, S, P are rendered in yellow, blue and red, respectively. b) Instantaneous configuration at *t* = 3321 where the C (red sphere) is across the edge of the stripe. c) Concentrations of species F and S along the *y* axis at *x* = 30. The width of the pattern is *W*_s_ = 5.5. The size of the system is *L*_*x*_ = 60, *L*_*y*_ = 30, and *L*_*z*_ = 20.

Our investigations of motor dynamics in systems with chemical patterns use a coarse‐grain microscopic description of the entire system. We consider sphere‐dimer motors^48^, ^49^ made from catalytic (C) and noncatalytic (N) spheres linked by a rigid bond that operate by a diffusiophoretic mechanism where chemical reactions on the catalytic sphere produce concentration gradients that drive propulsion. The solution in which the motors move contains fuel F, product P, and solvent S species whose local concentrations adopt the form of chemical patterns with various shapes. These species interact with the motors through intermolecular potentials and determine how the motors move in the presence of chemical gradients produced by the motors themselves and the chemical patterns. Pictures of the motor moving in a stripe chemical pattern are shown in **Figure** **1**a,b. Additional information concerning the motor and the construction of chemical patterns in the solution is given in the Experimental Section.

### 2.1 Single Motor Dynamics

The average velocity *V*_*u*_ of a motor along its bond may be computed from $V_{u}\;=\;〈V(t)\cdot \hat{u}(t)〉$, where $\hat{u}$ is the unit vector pointing from the N to C motor spheres. Here and below the angular brackets denote an average over time and different realizations of the motor dynamics. Since the orientation of a motor, $\hat{u}(t)$, in a homogeneous system experiences rotational Brownian motion with a characteristic relaxation time τ_*r*_, as well as self‐propulsion with velocity *V*_*u*_, its mean square displacement, Δ*L*(*t*)^2^, has early‐time ballistic behavior, $\Delta \;\;L{\left(t\right)}^{2}\;\sim \;V_{u}^{2}t^{2}$ and a long‐time diffusive behavior, Δ*L*(*t*)^2^ ∼ 6*D*_eff_ *t*, characterized by the effective diffusion coefficient, $D_{\mathrm{eff}}\;=\;D_{0}\;+\;\frac{1}{3}V_{u}^{2}\tau_{r}$, where *D*_0_ is the diffusion coefficient in the absence of propulsion. Rotational Brownian motion limits the ability of motors to carry out tasks that utilize their directed motion. Here we show that if the chemical pattern has some characteristic length that is of the same order as the motor size, rotational Brownian motion is reduced allowing it to effectively execute directed motion. Restriction to a domain does not occur through confining walls but through effects controlled by chemical gradients.

Consider the motion of a motor in a system with a chemical stripe of high fuel concentration with width *W*_s_ surrounded by a fuel‐poor region. A plot of the concentration profiles of the F and S species is shown in Figure 1c, and as color‐coded 2D representations in Figure 1a,b where an example of the motor dynamics is shown. If the motor is in the fuel‐poor phase it undergoes diffusive motion since the self‐propulsion mechanism does not operate. Encounter with the fuel‐rich stripe triggers the catalytic reactions F + C → P + C on the catalytic portion of the motor, which generates concentration gradients of the fuel and product species that lead to self‐propulsion. The subsequent dynamics of the motor in the stripe is shown in Figure 1a,b (see also Movie S1 in the Supporting Information.) The asymmetric distribution of P particles around the N sphere is shown at *t* = 744. Since we have chosen the interaction energies of the F and P species with the motor sphere N to satisfy ε_F_ > ε_P_, the motor will move with the C sphere as its head. Once the motor enters into the stripe the same forces that are responsible for self‐propulsion confine its dynamics to the stripe. Suppose the C sphere moves across the edge of the fuel‐rich stripe and a portion of this sphere enters the fuel‐poor domain. Then no chemical reaction will occur on this part of the catalytic surface, and a force asymmetry due to ε_S_ > ε_P_ will be produced on the C sphere that will tend to move it into the fuel‐rich region. One can see the asymmetric distribution of P particles around the C sphere that give rise to this effect in Figure 1b at *t* = 3321. Since no catalytic reactions occur on the N sphere and ε_F_ = ε_S_, the forces it experiences are due to the P concentration field produced by the C sphere.

**Figure 2:**
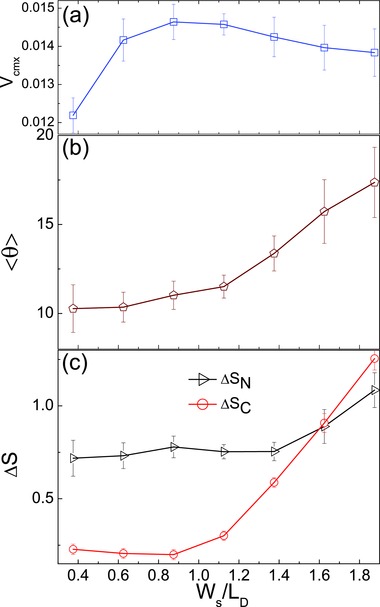
a) Plot of 〈*V*_*x*_〉 versus *W*_s_/*L*_D_. b) Plot of 〈θ〉 versus *W*_s_/*L*_D_. The data was obtained from time and ensemble averages over 20 realizations of the dynamics. c) Plot of Δ*S*_α_ versus *W*_s_/*L*_D_; Δ*S*_C_ (circles) and Δ*S*_N_ (triangles). The system size is *L*_*x*_ = 60, *L*_*y*_ = 30, and *L*_*z*_ = 20.

The ratio *W*_s_/*L*_D_, where *L*_D_ = 2σ_α_ is the dimer length, is an important factor that affects the character of the dynamics. As the width of the stripe increases the motor will no longer be strongly confined to move along the *x* axis. **Figure** **2**a shows that the average value of the *x*‐component of the motor velocity, 〈*V*_*x*_〉, is a nonmonotonic function of *W*_s_/*L*_D_. For values of *W*_s_ that are smaller than the diameter of the dimer spheres, only a portion of the C surface will be exposed to fuel and its velocity will be small. When the width of the stripe increases, so does the motor velocity due to the increased availability of fuel. This increase cannot continue because, as the width increases further the motor experiences orientational fluctuations that reduce 〈*V*_*x*_〉. The average angle the motor makes with the *x* axis, $〈\theta 〉\;=\;\left\langle \arccos(\hat{u}(t)\cdot \hat{x})\right\rangle$, is plotted in Figure 2b which shows that this angle is an increasing function of *W*_s_.

The effects of rotational fluctuations are shown in Figure 2c where $\Delta \;\;S_{\alpha }\;=\;\sqrt{〈{\left(y_{\alpha }(t)\;-\;L_{y}/2\right)}^{2}〉}$, the mean square deviation of the *y* component of the position of dimer sphere α = C, N from the center of the stripe. One can see that Δ*S*_C_ decreases slightly as *W*_s_/*L*_D_ increases, which indicates that the confinement of this sphere increases. The N sphere is free to rotate due to its lack of catalytic activity, so that Δ*S*_N_ > Δ*S*_C_. As the width of the stripe increases further there is a regime where Δ*S*_N_ < Δ*S*_C_. In this regime the interactions of the C sphere with edges of the stripe, where concentration gradients are large, induce active orientational changes as a result of forces on the C sphere.

**Figure 3:**
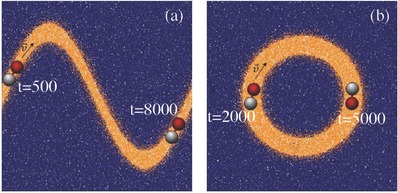
a) Dimer dynamics in a sinusoidal (“∼”) chemical pattern. The wave length of the pattern is *L*_*x*_/2 and its amplitude is *L*_*y*_/2. b) Dynamics in an annular “◯” chemical pattern with radius *r*_◯_ = 15. The motor is shown at two different times indicated in the figures. The system size is *L*_*x*_ × *L*_*y*_ × *L*_*z*_=60 × 60 × 20. The width of the patterns is *W*_s_ = 5.5.

**Figure 4:**
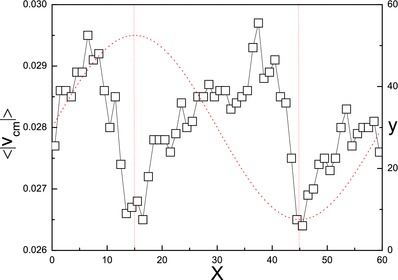
Plot of 〈|**V**_cm_|〉 versus position along the sinusoidal path. The dotted line is the sinusoidal function of the pattern. The average was obtained from time and ensemble averages over 30 realizations of the dynamics.

The fuel‐rich domain need not take the form of straight stripe and **Figure** **3** shows the dynamics of a motor in sinusoidal “∼” and annular “◯” domains. (also see Movies S2 and S3 in the Supporting Information.) As expected the motor is able to follow the paths prescribed by these chemical patterns but the pattern curvature will modify the dynamics. **Figure** **4** plots the average speed of the center of mass of the motor, 〈|**V**_cm_|〉, as function of position along the sinusoidal path, and shows that it varies with the spatial period of the path. As might be anticipated the motor slows when it approaches points of high curvature. The annular path has constant curvature and no such effect is seen although the mean speed is smaller than that in a straight stripe of similar width.

### 2.2 Collective Dynamics in Chemical Patterns

Often collections of active particles are observed to form dynamic clusters, segregate into low and high density domains, and evolve into other inhomogeneous states not seen in equilibrium systems.^20^, ^21^, ^22^, ^23^, ^24^ Simple active Brownian particle models are able to capture some aspects of this dynamics.^50^, ^51^, ^52^, ^53^, ^54^

Since propulsion of motors by self‐diffusiophoresis occurs through the generation of fuel and product concentration gradients, these motors can also respond to external gradients, including those produced by other motors. For example, if a motor tends to move toward high product concentrations, it will tend to move toward other motors that produce product leading to clustering in many‐motor systems. The collective dynamics of self‐diffusiophoretic motors present new features.^55^, ^56^, ^57^ Microscopic simulations of the collective behavior of Janus motors have shown that both chemical gradients and hydrodynamic effects are important in determining the nature of the observed inhomogeneous states.^58^ In addition, the collective dynamics of these motors is affected by how they are confined, how the nonequilibrium state is maintained and their shapes. For example, the dynamic clusters of sphere dimers with nonspherical shapes differ from those of spherical Janus motors.^59^, ^60^ Here we investigate some aspects of the collective behavior of small numbers of sphere dimers when confined to domains by forces determined by chemical gradients. We show that motors aggregate in specific spatial regions of chemical patterns. Thus, the collective behavior is controlled by self‐induced chemical gradients as well as gradients arising from chemical patterns.

**Figure 5:**
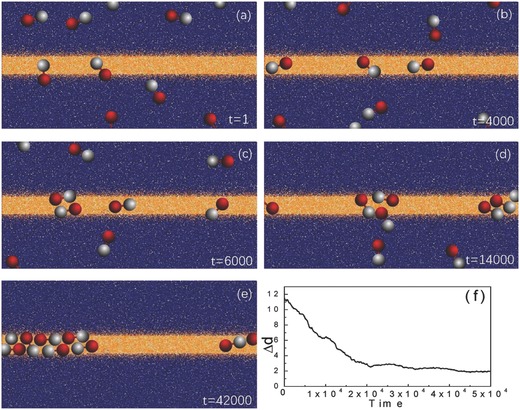
Dynamics of *N*_d_ = 8 sphere dimer motors in a system with a stripe chemical pattern. Panels (a)–(e) show instantaneous configurations of F (yellow) and S (blue) molecules. The P species molecules are not shown. Panel (f) shows the evolution of Δ*d* versus time for *N*_d_ = 8 averaged from 5 realizations. The system size is *L*_*x*_ = 80, *L*_*y*_ = 40, and *L*_*z*_ = 20.

**Figure** **5** gives an overview of the dynamics of *N*_d_ = 8 sphere dimer motors which are initially randomly distributed in a system with a stripe chemical pattern. Figure 5a shows an initial configuration where no C sphere lies completely within the fuel‐rich stripe. The mechanism by which a motor is captured by a stripe pattern (or fuel‐rich domains of other shapes) was discussed earlier for a single motor. Motors whose C spheres lie at the edge of or in the vicinity of the stripe are able to produce product P by motor catalytic reactions that allow them to chemotactically respond the pattern fuel gradient, while motors outside the fuel‐rich region undergo Brownian motion until they are able to respond to the fuel gradient. The remainder of the panels in the figure show instantaneous configurations in the course of the evolution leading to the final state in panel (e) where all motors are trapped within the stripe (see Movie S4 of the Supporting Information.) The capture process can be monitored by following the evolution of the average *y*‐component of the distance of the dimer center of mass from the center of pattern at *L*_*y*_/2

(1) $$\Delta \;\;d\;=\;\left\langle \frac{1}{N_{d}}\sum_{i=1}^{N_{d}}|y_{i}(t)\;-\;L_{y}/2|\right\rangle$$

where the angle bracket means an average over realizations. This quantity is plotted in Figure 5f versus time and shows the decay to the fully confined collective state.

**Figure 6:**
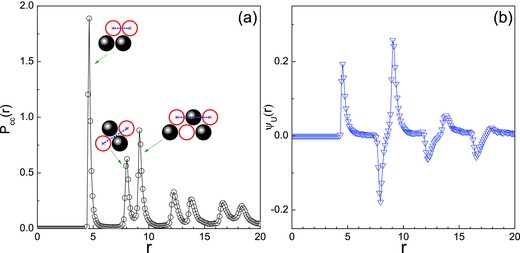
Plots of the probability density function *P*_CC_(*r*) scaled by *L*_*x*_ a) and the spatial orientational correlation function ψ_*u*_(*r*) b). The insets in panel (a) show configurations, with corresponding dotted lines indicating distance, contributing to the peaks in the probability density function. The parameters are the same as those in Figure 5. The C and N monomers are indicated by open and full circles, respectively.

As the motors accumulate in the stripe they form dynamic clusters, subject to thermal fluctuations where motors leave and enter the clusters, eventually reaching a statistically stationary regime, which can be characterized by the steady‐state correlations of motor positions and orientations. **Figure** **6** shows a plot of the probability density of C–C distances

(2) $$P_{\mathrm{CC}}(r)\;=\;\frac{1}{N_{C}^{2}}〈\sum_{j<i=1}^{N_{C}}\delta (|(r_{i}\;-\;r_{j})|\;-\;r)〉$$

where **r**_*i*_ is the position of catalytic monomer *i*, *N*_C_ = *N*_d_ is the number of catalytic monomers. There are well defined peaks in this function that can be identified with the configurations in the inset, indicating that these structures persist throughout the course of the steady‐state evolution. Similar correlations are observed in C–N and N–N probability density functions.

The local orientational order can be described by the function

(3) $$\psi_{u}(r)\;=\;〈\frac{1}{n(r)}\;\sum_{j<i=1}^{N}({\hat{u}}_{i}\cdot {\hat{u}}_{j})\;\delta \;(|(r_{i}\;-\;r_{j})|\;-\;r)〉$$

where $n(r)\;=\;\sum_{j<i=1}^{N}\delta \;(|(r_{i}\;-\;r_{j})|\;-\;r)$ is the number of motor pairs separated by the distance *r*. From this definition it follows that two motors separated by the distance *r* have the same or opposite orientations when ψ_*u*_(*r*) > 0 or ψ_*u*_(*r*) < 0, respectively, whereas ψ_*u*_(*r*) = 0 indicates that there is no preferential orientation for the motor pair. The fact that the first peaks in the ψ_*u*_(*r*) and *P*_CC_(*r*) functions are at approximately the same value of *r*, ψ_*u*_(*r*) > 0 signals that motors with two neighboring C–C monomers tend to align with each other, indicted by the configuration in the inset of Figure 6a. The first negative peak in ψ_*u*_(*r*) corresponding to the second peak in *P*_CC_(*r*) signals antiparallel alignment of two neighboring dimers. Other long‐range positional ordering, e.g. the third peak in *P*_CC_(*r*), is also characterized distinctive orientational order in ψ_*u*_(*r*).

**Figure 7:**
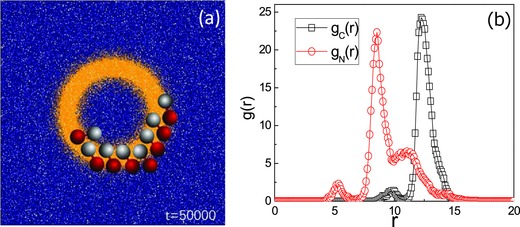
a) An instantaneous configuration drawn the dynamical evolution of an ensemble of *N*_d_ = 8 dimer motors within an annular region with inner radius *r*_in_ = 8.0 and outer radius *r*_out_ = 12. b) Radial distribution function *g*(*r*) of the N and C monomers for the system.

The change from a stripe to an annular pattern leads to changes in the collective behavior when all other conditions are the same. While dynamic clusters are again formed, their structures are different (see Movie S5 in the Supporting Information). A configuration drawn from the dynamics of an ensemble of *N*_d_ = 8 dimers is shown in **Figure** **7**a. A characteristic feature of such configurations is that most of the motors point with their catalytic heads outward, i.e., in the outer edge of the annulus, while the *N* monomers lie in the inner edge. The generality of this feature is confirmed by the structure of the radial distribution function *g*_α_(*r*)

(4) $$g_{\alpha }(r)\;=\;\frac{1}{2\pi rn}\;〈\sum_{i=1}^{N}\delta ((|r_{\alpha ,i}-r_{C}|)\;-\;r)〉$$

where **r**_α, *i*_ is the position of monomer *i* of species type α, **r**_C_ the position vector of the center of the annular pattern, and *n* the number density of dimers in the confining plane. These functions are plotted in Figure 7b. One can see that each of these functions for α = C, N have a dominant peak. The *g*_N_(*r*) function has a large peak at *r* ≃ 8.5, which is slightly greater than the inner annulus radius *r*_in_ = 8.0, while *g*_C_(*r*) has a large peak at *r* ≃ 12.2, indicating that the C monomers are distributed around the outer edge of annular pattern at *r*_out_ = 12.

**Figure 8:**
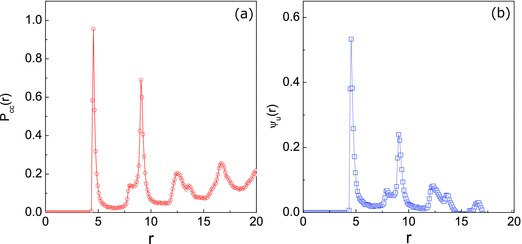
a) Plots of the probability density, *P*_CC_(*r*), and b) spatial orientational correlation, ψ_*u*_(*r*), functions.

The steady state structural correlations of the clusters in the annulus are evident in the *P*_CC_(*r*) and ψ_*u*_(*r*) functions plotted in **Figure** **8**a,b, respectively. The first peak in *P*_CC_(*r*) again appears at the nearest neighbor CC distance, while ψ_*u*_(*r*) > 0 at this distance indicates that the neighbor dimer pair tends to align. However, in contrast to the stripe results in Figure 6a, the structures of the other peaks are different and in Figure 6b one see that ψ_*u*_(*r*) > 0 indicating long‐range orientational order.

**Figure 9:**
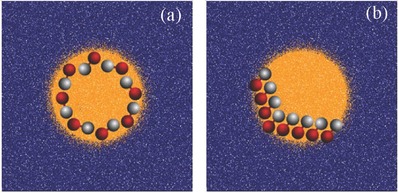
Instantaneous configurations at *t* = 32500 a) and *t* = 48000 b) plotted from the dynamical evolution of an ensemble of *N*_d_ = 8 dimer motors in an disc region with radius *r*_disc_ = 12.0.

Finally, as an example of a chemical pattern with all dimensions larger than a dimer we show the dynamics of *N*_d_ = 8 in a disc of high fuel concentration. Through Brownian motion, dimer distributed throughout the system will reach the disc and be trapped there. The motors generate high concentrations of product in the disc interior and since ε_P_ < ε_S_ they tend to remain confined within the disc. The resulting statistically steady state dynamics shows the existence of dynamic clusters of various types (see **Figure** **9** and Movie S6 in the Supporting Information). An examination of many realizations of the dynamics indicates that two cluster types predominate. The dominant clusters are similar to those of the annulus but there are also transitions to and from states where the dimers form a ring near the perimeter of the disc. This example shows how confinement by the pattern can influence active self‐assembly. One can also see that a number of factors, including the size of the domain, the density or volume fraction of motors, and the interaction potentials, will determine the nature of the self assembled structures.

## 3 Conclusion

Chemically propelled motors require fuel supplied under nonequilibrium conditions for their operation. The medium in which motors move may not simply provide a source of fuel that is used in catalytic reactions on the motor that lead to motor propulsion. Instead, the surrounding medium may itself contain species that participate in chemical reaction networks that also operate out of equilibrium and may undergo bifurcations to inhomogeneous stationary and dynamic states. The nonequilibrium states have spatially varying chemical composition, often comprising domains separated by strong gradients of chemical species. The simulations in this study pointed to some of the phenomena one may expect when diffusiophoretic motors operate in chemically patterned media where fuel and product species participate in the bulk phase far‐from‐equilibrium reactions. Because the domains are not formed by confining walls, wall effects on hydrodynamic flows do not play a role, and motor confinement is due to chemotactic effects. However, one may also consider surfaces partially coated with catalysts with specifically designed geometries that produce high (or low) fuel concentrations. Motors will then be able to interact with such catalytic patterns through the mechanisms described in this paper.

When some characteristic lengths of the pattern are comparable to those of the motor we showed that rotational Brownian motion can be inhibited so that the motor is able navigate along the pattern. For micrometer‐scale motors chemical patterns with suitable length scales could be produced in systems with competing interactions or microfabricated catalytic structures.

It is much more common for chemical patterns in nonlinear reaction‐diffusion systems to have much larger length scales, and these patterns can also be exploited to study aspects of the collective motions of chemically powered motors in far‐from‐equilibrium systems. The larger domains in these systems can act as sinks that attract or accumulate motors which then behave collectively, confined only by chemical gradients. In this context, chemical patterns could be used to promote specific types of active self‐assembly for use in the design of new materials. The full range of motor collective behavior in such systems has not been investigated and would add to our knowledge of the collective dynamics of active particles.

One motivation for this research was to understand, in a general context, how chemically powered motors, either singly or collectively, move in complex chemical media that are maintained out of equilibrium and support inhomogeneous spatiotemporal states. Our study addressed only simple aspects of this topic but the simulation techniques used here are easily generalized to include reaction networks corresponding more realistic situations that will permit such investigations to be carried out.

## 4 Experimental Section

The simulations of the dynamics of systems containing sphere‐dimer motors and a large number of solvent molecules were carried out using hybrid MD‐MPC dynamics.^61^, ^62^ The sphere‐dimer motor was propelled by a self‐diffusiophoretic mechanism in a far‐from‐equilibrium reacting medium with chemical patterns. The motor and all chemical species in the surrounding medium were contained in a 3D box with a slab geometry and confined between two parallel walls at a distance *L*_*z*_ apart, perpendicular to the *z* direction of the system. Periodic boundary conditions were applied in the *x* and *y* directions with dimensions *L*_*x*_ and *L*_*y*_, respectively.

*Sphere‐Dimer Motor and Solvent*: Sphere‐dimer motors were made from catalytic C and noncatalytic N spheres separated by a bond distance *R* that was fixed by a holonomic constraint.^48^ Each sphere in the dimer interacted with the walls of the slab through a 9‐3 Lennard‐Jones (LJ) potential, $V_{\mathrm{LJ}}^{93}(r)\;=\;\varepsilon_{w}[{\left(\sigma_{w}/r\right)}^{9}\;-\;{\left(\sigma_{w}/r\right)}^{3}]$, where ε_w_ and σ_w_ are the wall energy and distance parameters, respectively. The motor executes quasi‐two‐dimension motion in the *x*–*y* plane as a result of the sphere–wall interactions.

The chemically active solution contains *N* = *N*_F_ + *N*_P_ + *N*_S_ point‐like fuel F, product P, and solvent S particles with identical masses *m*_s_ and concentrations *c*_I_, I = F, P, S. These particles interact with the walls in the *z* direction through bounce‐back collisions that reverse their velocity after collision. They interact with the dimer spheres through repulsive Lennard‐Jones interactions, *V*_αI_(*r*) = 4ε_αI_[(σ_α_/*r*)^12^ − (σ_α_/*r*)^6^ + 1/4]Θ(*r*_*c*_ − *r*), where Θ is a Heaviside function and *r*_c_ = 2^1/6^σ_α_ is the cutoff distance, with α = C, N. The labels α and *I* are used to denote the various interactions between the species in the solution and dimer spheres. The interactions with the C and N spheres were chosen to be the same (interactions are independent of α) so that the notation was simplified to *V*_αI_ = *V*_I_ and ε_αI_ = ε_I_. In the studies of collective motion there were repulsive Lennard‐Jones interactions between monomers on different dimers with ε_D_ and σ_D_.

Chemically powered motors that operate by a self‐diffusiophoretic mechanism use chemical reactions F + C → P + C on the catalytic portion of the motor to generate concentration gradients of the fuel and product species that lead to a fluid slip velocity on the outer edge of the boundary layer around the motor from which the velocity of the motor can be determined.^63^, ^64^, ^65^ The nonequilibrium state was maintained in the microscopic simulations as follows: when P particles diffuse a distance *r*_P_ from a dimer they are converted back to F by the bulk reaction P → F.

*Chemical Patterns*: In continuum models, chemical patterns were obtained from solutions of reaction–diffusion equations constructed from chemical networks operating under nonequilibrium conditions. The nonlinear chemical rate equations, the rate coefficients that enter these equations, and the values of the diffusion coefficients of the chemical species determine the forms that the chemical patterns take.^32^, ^33^ Microscopic or mesoscopic models instead describe the system at particle level and the chemical spatial structures arise from the reactive collision processes that underlie the chemical rate laws, along with the random diffusive motions of the molecules.

The inhomogeneous states that occur in reactive systems driven far from equilibrium comprise spatial regions with different chemical composition often separated by sharp chemical gradients. In order to study some aspects of the interaction of self‐diffusiophoretic sphere‐dimer motors with chemical patterns it was sufficient to use a simple microscopic reaction model designed to yield chemical domains with various shapes. Reaction mechanisms that give rise to bifurcations leading to pattern formation were studied from mesoscopic perspective using reactive multiparticle collision dynamics.^66^ A domain of arbitrary shape containing a high concentration of fuel molecules in a “sea” of a low‐fuel phase may be constructed as follows: when molecules diffused into the domain they were converted to F particles; when molecules diffused out of the domain they undergo the reaction, F (or $P)\overset{k_{\mathrm{cat}}}{\to }S$, which converted them to S‐type particles with rate constant *k*_cat_. If the molecules diffuse out of the domains at time *t*, the time of the conversion to S at *t* + *t*_d_, can be computed from *t* + ln (1/*r*_1_)/*k*_cat_, where *r*_1_ is random number chosen from a uniform distribution on the interval [0,1). Although the domain constructed in this way was not the result of a symmetry‐breaking bifurcation of the homogeneous state, the final inhomogeneous state had the main characteristics of chemical patterns arising from Turing bifurcations or those in bistable media with competing interactions, namely, domains of high and low concentrations of chemical species separated by sharp chemical gradients. In Figure 1a,b, the color‐coded 2D stripe‐shaped patterns with width *W*_s_ are shown, and corresponding concentration profiles of the F and S species are plotted in Figure 1c. The stripe pattern extended across the simulation box in the *z* direction between the confining walls. The figure also shows two instantaneous configurations of a sphere‐dimer motor in the system.

*System Dynamics*: The evolution of the system was carried out using hybrid molecular dynamics‐multiparticle collision dynamics. In multiparticle collision dynamics, fictitious solvent particles, representing coarse‐grained real molecules, streamed and underwent effective collisions at discrete time intervals *t*_MPC_, which accounted for the effects of many real collisions during this time interval. Descriptions of the simulation method can be found in refs. 61, 62, 67, 68. In the streaming step Newton's equations of motion were integrated for all interacting particles. Since reactions occur in the bulk of the solution and are responsible for maintaining the chemical pattern, the reactive version of multiparticle collision dynamics was employed for particles in the solution.^66^ This microscopic dynamics was able to describe the concentration and fluid flow fields that were part of the diffusiophoretic propulsion mechanism, as well as thermal fluctuations. The simulation results presented below were reported in dimensionless units based on energy ε, mass *m*_s_, and multiparticle collision cell length *a*_0_. The time is in units of $t_{0}\;=\;{\left(m_{s}a_{0}^{2}/\varepsilon \right)}^{1/2}$, and temperature is reported as *k*_B_ *T*/ε. The velocity Verlet algorithm was used to integrate Newton's equations of motion with time step *t*_MD_ = 0.01. Multiparticle collisions using rotations by an angle θ_M_ = π/2 about a randomly chosen axis were carried out with *t*_MPC_ = 1.0. The average number of solvent molecules per cell is *n*_0_ = 10. The parameters in LJ potentials are ε_w_ = 5.0, σ_w_ = *L*_*z*_/2, σ_α_ = 2.0, ε_F_ = ε_S_ = 5.0, and ε_P_ = 0.1. For interactions between monomers on different dimers ε_D_ = 5.0 and σ_D_ = 4.2. The P → R conversion distance is *r*_P_ = 2σ_C_ + *R* = 8.6. System temperature is *T* = 1/6. Other parameters are: *k*_cat_ = 0.08, and *R* = 4.6. The masses of the C and N monomer were chosen according to their volumes to ensure that the dimer was approximately neutrally buoyant.

## Conflict of Interest

The authors declare no conflict of interest.

## Supporting information

Supplementary

Supplementary

Supplementary

Supplementary

Supplementary

Supplementary
